# Dual Effects of Hearing Loss and Dementia on Functional Limitations in Older Adults

**DOI:** 10.1093/geroni/igaa057.690

**Published:** 2020-12-16

**Authors:** Michael McKee, Yunshu Zhou, Joshua Ehrlich, Elham Mahmoudi, Jennifer Deal, Nicholas Reed

**Affiliations:** 1 University of Michigan, Ann Arbor, Michigan, United States; 2 Johns Hopkins Bloomberg School of Public Health, Baltimore, Maryland, United States; 3 Johns Hopkins University, Baltimore, Maryland, United States

## Abstract

Age-related hearing loss (HL) is both common and associated with elevated risk for cognitive decline and poorer health. To care for an aging population, it is critical to understand the effect of coexisting HL and dementia on functional activities. The effect of co-existing dementia and self-reported HL on daily functioning were assessed. A cross-sectional analysis was performed using nationally-representative data from the 2015 National Health and Aging Trends Study consisting of U.S. adults 65+. The sample included 1,829 adults with HL (22.8%) and 5,338 adults without HL. Multivariable Poisson regression was used to model the independent effects and interaction of self-reported HL and dementia status on three validated functional activity scales (self-care, mobility, and household). All analyses adjusted for sociodemographic and medical factors. HL participants were more likely to be white, older, male, less educated (p <0.01). 8.4% had possible dementia and 6.5% had probable dementia. Respondents with HL or possible or probable dementia had significantly lower mobility, self-care, and household activity scores (p<.001 for all comparisons) compared to their peers. A small yet significant interaction was present in all models, suggesting that HL respondents with co-occurring dementia had lower mobility, self-care, and household activity scores than predicted by the independent effects of dementia and self-reported HL (p<.001 for all comparisons). Older adults with co-occurring dementia and HL are at increased risk for poor functioning and should be screened by healthcare providers. Future work should consider the impact of intervention in this vulnerable/at-risk population.

